# Novel Omics Studies on Anisakid Nematodes

**DOI:** 10.3390/genes12081250

**Published:** 2021-08-16

**Authors:** Serena Cavallero, Fabrizio Lombardo, Stefano D’Amelio

**Affiliations:** Department of Public Health and Infectious Diseases, Sapienza University of Rome, 00185 Rome, Italy; serena.cavallero@uniroma1.it (S.C.); fabrizio.lombardo@uniroma1.it (F.L.)

Parasitic nematodes infecting humans and animals are widely distributed in marine and terrestrial environments, causing considerable morbidity and mortality globally. In fact, most parasitic nematodes are etiological agents of neglected tropical diseases (NTDs) and contribute to the global burden of such diseases to a degree of 4443.47 thousand disability-adjusted life years (DALYs). Among several families, anisakids are responsible for a relatively poorly known food-borne zoonosis called anisakidosis that results from the accidental ingestion of marine products harboring infective third-stage larvae (L3).

Despite the economic and medical impact of the diseases caused by these nematodes, advances towards understanding the biological mechanisms of infection, discoveries of new therapeutic agents, and developments of effective treatments are still limited. Progress has been hindered by the lack of funds dedicated, the absence of reliable genomic data for comparisons, and the intrinsic complexity of parasitic helminths compared to their closely related models or free-living organisms.

This Special Issue consists of seven original research articles, one review and one communication. These contributions synthesize fascinating novel approaches currently explored in several laboratories worldwide and provide a rare opportunity to illustrate a general overview of the ongoing projects in the field. All the manuscripts aimed to contribute knowledge on poorly investigated aspects on anisakids, taking advantage from modern and cutting-edge sequencing technology and bioinformatics. In particular, the implementation of high-throughput next-generation sequencing methods is assisting the spread of studies that, combined with advances in computational biology and bioinformatics, have greatly accelerated discoveries within basic and applied research for many parasitic diseases. These more general aspects are described in the review by D’Amelio et al. [1], where the most recent genomic, transcriptomic and proteomic studies dealing with biologically relevant aspects of anisakids are summarized.

The use of molecular tools to identify pathogens causing infectious zoonotic diseases in humans at species level is of particular interest. The communication by Roca-Geronès and colleagues aimed to characterize the etiological agent of anisakiasis in Spain, where a high number of cases have been recorded in recent years, mainly after the consumption of traditional culinary preparations based on raw or marinated marine products, although no species-specific diagnosis supported by molecular tools has been documented in humans thus far [2]. In this study, three patients who had consumed undercooked hake reported epigastric pain, and larval nematodes removed endoscopically from their stomachs were identified at a molecular level as *Anisakis simplex* sensu stricto, using a combined approach based on enzymatic digestion of the ribosomal nuclear region ITS and sequencing of a partial region of the subunit α of the elongation factor gene. This report highlights the importance of using proper molecular tools to obtain an accurate diagnosis of an infective disease. In fact, *Anisakis pegreffii* is considered the most common source of infection for Mediterranean cases, but its sibling species *A. simplex* s.s. may also be observed.

Aspects related to host–pathogen interactions are fascinating, but very few studies address this topic in the field of anisakidosis. A pivotal contribution from Mladineo and Hrabar [3] highlights the effect of parasite stimulus on the structure of the leukocyte nucleolus. Using confocal microscopy, they demonstrated that crude *A. pegreffii* extract triggered structural changes in leukocyte nucleoli as early as 12 h post-parasitic stimuli. Such observations may be explained by a change in ribosynthesis, one of the cell strategies to maintain homeostasis under stress conditions. A similar background inspired the research article of Palomba et al. [4], who aimed to investigate gene expression profiles in the nematode larval stage during the infection of a natural host (fish), when the third-stage larvae tend to encapsulate in different tissues. Transcriptional modulation of genes encoding for proteins of interest, such as glycoproteins or metallopeptidases, may promote the understanding of parasitic biological traits related to their adaptive ability to survive within the hosts.

A comparative transcriptomic approach was undertaken by Cavallero et al. to characterize gene expression and gene products related to the pharyngeal portion, a key organ of the infective larval stage particularly rich in secretory gland cells, which is involved in pathogenic mechanisms [5]. Transcripts specifically enriched in the pharynx were analyzed in the two main etiological agents of anisakiasis, namely, *A. simplex* s.s. and *A. pegreffii*, and in a third marine parasite that is widespread in fish, namely, *Hysterothylacium aduncum.* Despite *H. aduncum* having rarely been associated with human disease, it is still listed as a zoonotic parasite. However, results from this study provided the first clues aiming to explain the differences in pathogenicity among these parasitic species, suggesting that *H. aduncum* should be considered of negligible concern for public health.

The other four research articles describe different proteomic approaches. Thanks to MALDI-TOF/TOF analyses, Robertson and colleagues [6] screened the immunoreactive properties of gland cell proteins using the sera of *Anisakis* spp.-allergic patients. This investigation revealed the presence of specific *Anisakis* antigens as well as secreted molecules (among them: peptidases, lipase-like and chitinases) shared with phylogenetically related parasitic nematodes. The second proteomics research article by Arcos and collaborators aimed to compare proteomes obtained from different members of *A. simplex* sensu lato complex (*A. simplex* s.s., *A. pegreffii* and their hybrid) [7]. This comparison revealed thirty-seven proteins as discriminant taxonomic biomarkers, most of which exhibited crucial biological functions related to allergy, innate immunity and interactions with the host. Another interesting proteomics study performed by Marzano et al. [8] aimed to use MALDI–TOF MS profiling and LC–ESI–MS/MS to obtain a fingerprint of the pathogen, with the view of constructing the first spectral library for the diagnosis of *Anisakis* infections to support clinical laboratories. The most promising signals evaluated as a potential diagnostic tool were associated with proteins originating in the ribosomal fraction. Finally, the manuscript from Polak and colleagues [9] aimed to investigate the role of anthelmintics on invasive larvae of *A. simplex* s.s, analyzing, for the first time, the underlying molecular mechanisms. In particular, new insights in the specific field were provided by tandem mass tag (TMT) labeling and extensive liquid chromatography coupled with tandem mass spectrometry (LC-MS/MS) analysis used to study the effect of Ivermectin on the proteome of *A. simplex* s.s. in vitro.

All the manuscripts included in this Special Issue contributed to filling gaps in the knowledge of fundamental aspects of anisakiasis, providing the scientific community with new, reliable and comprehensive datasets, and evidence based on the genomics, transcriptomics and proteomics of anisakids. As with many other parasitic nematodes, most of the subtle molecular mechanisms of infectivity, parasitic behavior and responses to treatment are still poorly investigated, and further studies will therefore benefit from these datasets which are now available.

## Data Availability

Not applicable.
